# Radiotherapy Response Patterns in Thoracic NUT Carcinoma: A Case Report

**DOI:** 10.1002/rcr2.70475

**Published:** 2026-01-14

**Authors:** Fumihiro Kashizaki, Nanami Tsuchiya, Shohei Watanabe, Hanming Lin, Ryusuke Orii, Kentaro Yumoto, Yoshiyuki Yasuura, Naomi Kawano, Hiroyuki Osawa, Harumi Koizumi, Kenichi Takahashi

**Affiliations:** ^1^ Department of Respiratory Medicine Yokohama Minami Kyosai Hospital Yokohama Japan; ^2^ Department of General Thoracic Surgery Yokohama Minami Kyosai Hospital Yokohama Japan; ^3^ Department of Pathology Yokohama Minami Kyosai Hospital Yokohama Japan

**Keywords:** bone metastasis, nuclear protein in testis (NUT) carcinoma, radiotherapy, superior vena cava syndrome

## Abstract

NUT carcinoma is a rare and highly aggressive malignancy, particularly when arising in the thorax. Radiotherapy is commonly used for symptom palliation; however, radiotherapeutic response patterns in thoracic NUT carcinoma remain poorly characterised. We report a 22‐year‐old woman with thoracic NUT carcinoma who demonstrated markedly heterogeneous responses to palliative radiotherapy across metastatic sites. A thoracic lesion causing superior vena cava (SVC) syndrome showed sustained radiographic improvement after irradiation, whereas a pelvic bone metastasis progressed shortly after single‐fraction radiotherapy despite transient symptom relief. Although differences in delivered radiation dose likely contributed to these outcomes, this case illustrates practical considerations in palliative radiotherapy for thoracic NUT carcinoma, including effective symptom control for SVC syndrome and the limited durability of single‐fraction radiotherapy for bone metastases. This case highlights practical considerations in palliative radiotherapy for thoracic NUT carcinoma and provides an educational perspective for respiratory physicians involved in multidisciplinary cancer care.

## Graphical abstract

We report a young woman with thoracic NUT carcinoma who experienced differential clinical responses to palliative radiotherapy across metastatic sites during the same clinical course. Rather than establishing intrinsic differences in radiosensitivity, this case illustrates real‐world clinical challenges in the management of advanced thoracic NUT carcinoma, including the need for emergent radiotherapy for superior vena cava syndrome prior to definitive diagnosis and the limited durability of single‐fraction radiotherapy for bone metastasis–related pain.

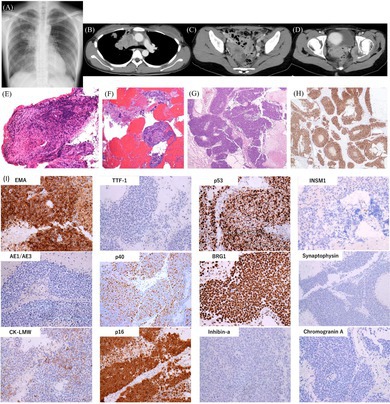

## 1 Introduction

NUT carcinoma is a rare and highly aggressive epithelial malignancy characterised by chromosomal rearrangements involving *NUTM1* [1]. Owing to its extreme rarity and nonspecific clinical presentation, diagnosis is often delayed, and prognosis remains poor, particularly when the disease arises in the thorax [1]. Radiotherapy is frequently employed for symptom palliation in advanced cases; however, radiotherapeutic response patterns in thoracic NUT carcinoma have not been well characterised.

We report a young woman with thoracic NUT carcinoma who experienced differential clinical responses to palliative radiotherapy across metastatic sites during the same clinical course. Rather than establishing intrinsic differences in radiosensitivity, this case illustrates real‐world clinical challenges in the management of advanced thoracic NUT carcinoma, including the need for emergent radiotherapy for superior vena cava (SVC) syndrome prior to definitive diagnosis and the limited durability of single‐fraction radiotherapy for bone metastasis–related pain.

## 2 Case Report

A 22‐year‐old woman with no significant medical history and a 0.5 pack–year smoking history presented with progressive right shoulder pain lasting 5 months, followed by left buttock pain. She was initially evaluated by gynaecology and orthopaedics for a suspected ovarian tumour; however, chest radiography incidentally revealed a right lung mass, prompting referral to our department.

**FIGURE 1:**
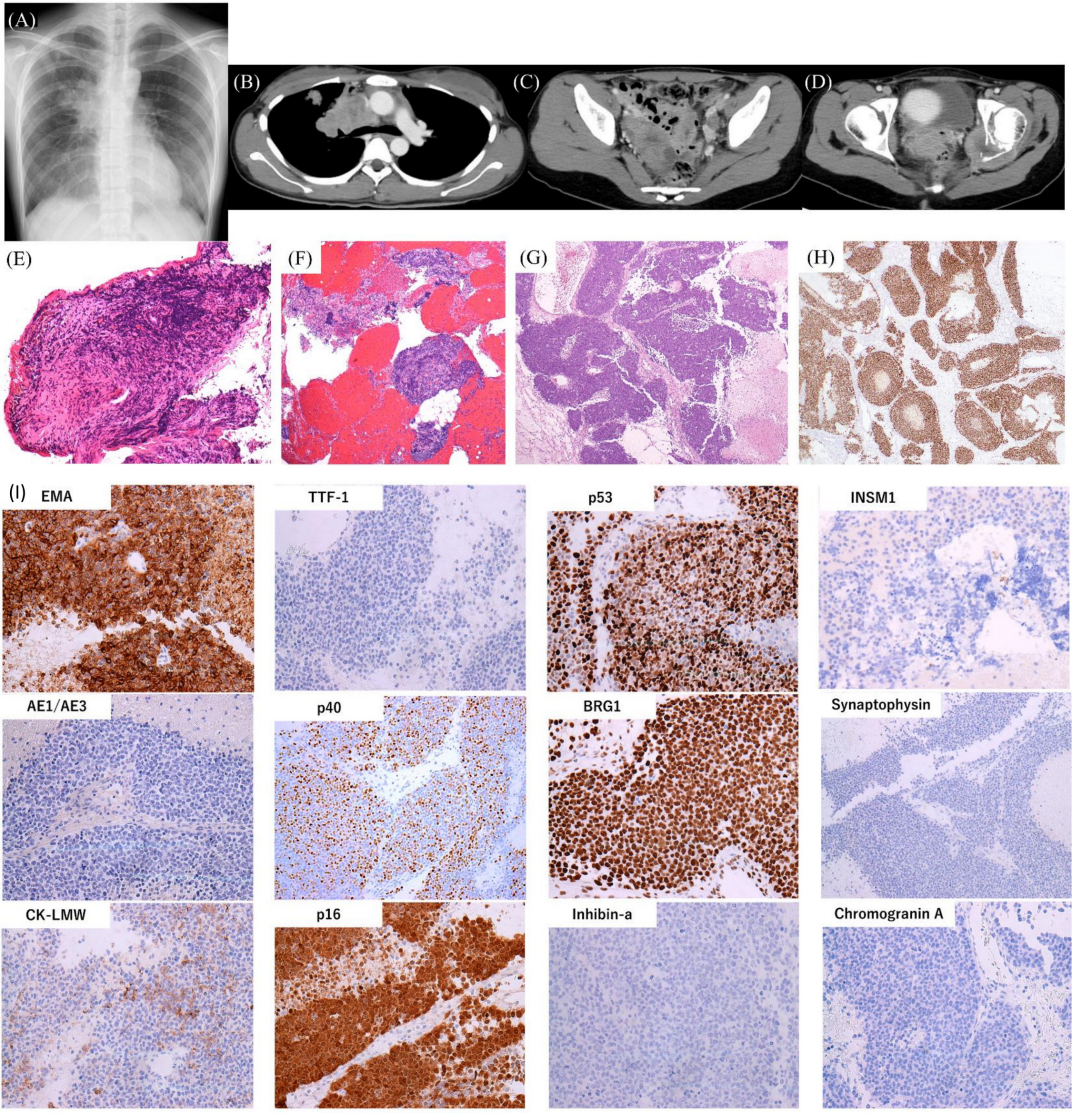
Radiographic findings and diagnostic histopathology of thoracic NUT carcinoma. (A) Chest radiograph showing a 20‐mm right upper lung nodule and a 50‐mm right hilar mass. (B) Chest computed tomography (CT) demonstrating a right S3 nodule contiguous with a hilar soft‐tissue mass compressing the superior vena cava (SVC). (C) Contrast‐enhanced CT revealing a 70‐mm heterogeneous soft‐tissue mass in the pelvis. (D) Pelvic CT showing a soft‐tissue mass with osteolytic destruction of the left ischium, consistent with bone metastasis. (E) Haematoxylin–eosin (H&E) staining of the transbronchial biopsy specimen demonstrating sheets of undifferentiated tumour cells with hyperchromatic round‐to‐oval nuclei and extensive necrosis. (F) Endobronchial ultrasound–guided transbronchial needle aspiration (EBUS‐TBNA) specimen showing similar undifferentiated tumour cells. (G) H&E staining of the laparoscopically resected right ovarian mass demonstrating sheets of undifferentiated tumour cells. (H) Immunohistochemistry showing strong nuclear NUT positivity, confirming the diagnosis of NUT carcinoma. (I) Representative immunohistochemical findings other than NUT, supporting the diagnosis of thoracic NUT carcinoma.

Chest radiography demonstrated a 20‐mm right upper lung nodule and a 50‐mm right hilar mass (Figure 1A). Computed tomography (CT) revealed a right S3 nodule contiguous with a hilar soft‐tissue mass compressing the SVC (Figure 1B), as well as a 70‐mm pelvic soft‐tissue mass (Figure 1C) with osteolytic destruction of the left ischium (Figure 1D), suggesting distant metastasis.

Laboratory testing showed elevated lactate dehydrogenase, C‐reactive protein, calcium, CA125 and neuron‐specific enolase. Transbronchial biopsy (Figure 1E) and endobronchial ultrasound–guided transbronchial needle aspiration (Figure 1F) were performed, and laparoscopic resection of a right ovarian mass (Figure 1G) was undertaken. Histopathological examination revealed sheets of undifferentiated tumour cells with extensive necrosis (Figure 1E–G). Immunohistochemistry showed positivity for epithelial membrane antigen, p53, p16 and BRG1, with focal cytokeratin expression, while neuroendocrine markers were negative (Figure 1I). A provisional diagnosis of undifferentiated carcinoma was made, and a second pathological review was requested.

**FIGURE 2:**
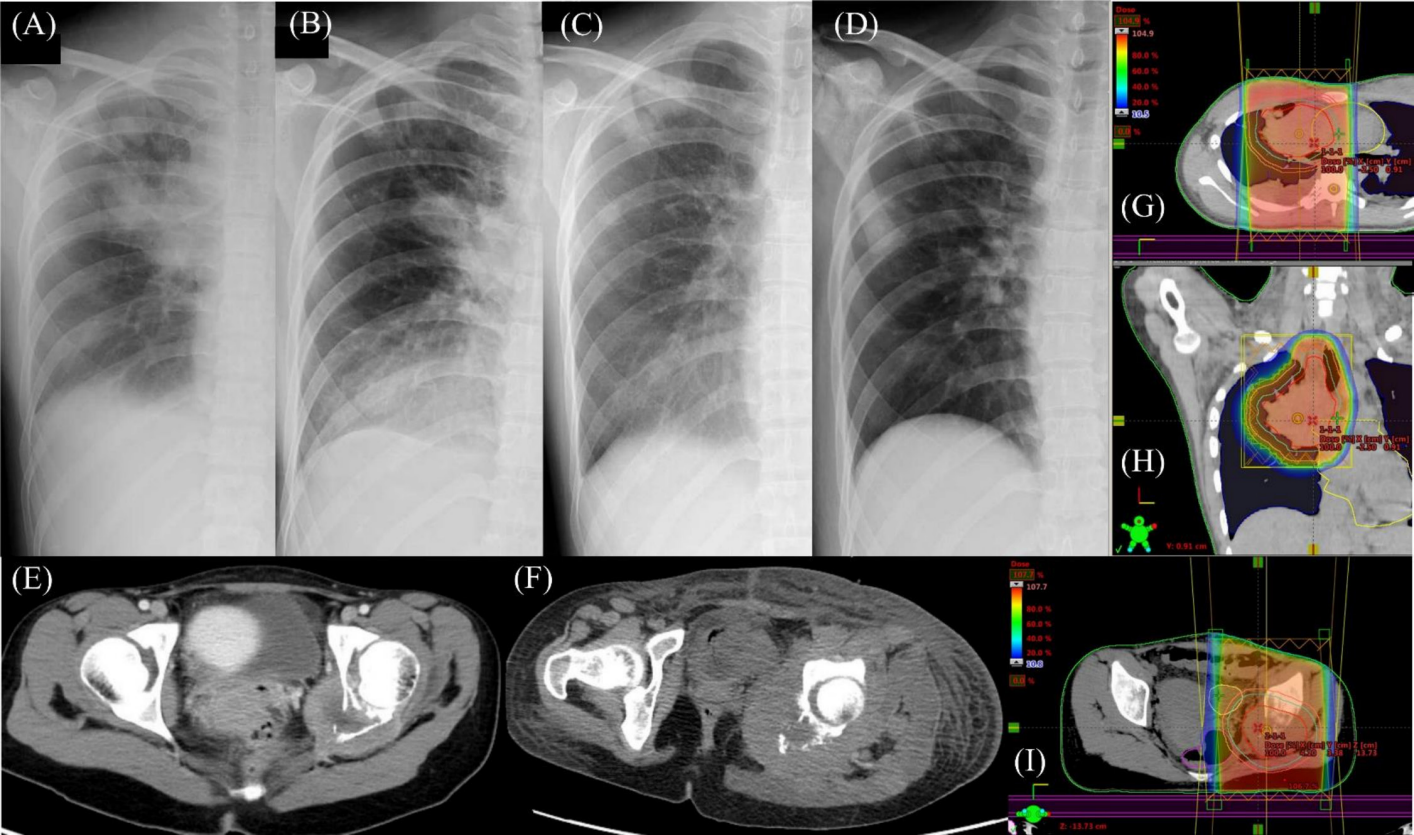
Radiographic course and radiotherapy planning CT findings. (A) Facial oedema and headache due to SVC syndrome improved promptly after thoracic palliative radiotherapy (20 Gy in 5 fractions). (B, C) The right hilar mass showed marked shrinkage at 1 week (B) and sustained reduction at 1 month (C) after thoracic irradiation. (D) At 16 weeks, a new right lateral pulmonary recurrence developed in a region that had received a substantially lower radiation dose than the high‐dose hilar field. (E, F) In contrast, the left pelvic bone metastasis treated with single‐fraction radiotherapy (8 Gy in 1 fraction) showed radiographic progression by 16 weeks, despite transient symptom relief. (G, H) Axial (G) and coronal (H) radiotherapy planning CT images demonstrating the dose distribution for thoracic irradiation, with tumour response localised to the higher‐dose region. (I) Radiotherapy planning CT image for the pelvis showing the dose distribution surrounding the left ischial lesion. Images in panels (B–F) were obtained during the same terminal phase of the disease, allowing direct comparison of radiographic response within and outside the thoracic radiation field and at the pelvic metastatic site.

During diagnostic evaluation, the patient developed progressive facial edema and headache consistent with SVC syndrome. Urgent palliative radiotherapy was therefore delivered to the thoracic lesion (20 Gy in 5 fractions) (Figure 2G,H), followed by single‐fraction radiotherapy to the left ischial lesion (8 Gy) for pain control (Figure 2I). Symptoms related to SVC obstruction improved promptly (Figure 2A–C) and were sustained (Figure 2D), whereas the pelvic lesion showed only transient pain relief and radiographic progression within weeks (Figure 2E,F).

The second pathological review demonstrated strong nuclear NUT immunoreactivity (Figure 1H,I), confirming the diagnosis of NUT carcinoma. Comprehensive genomic profiling revealed low tumour mutational burden and no *BRD4–NUTM1* rearrangement. Combination chemotherapy with pembrolizumab, carboplatin and nab‐paclitaxel was initiated; however, disease progression occurred in non‐irradiated pulmonary lesions and the pelvic metastasis. The patient died 133 days after admission.

## 3 Discussion

In this case, palliative radiotherapy was prioritised to manage life‐threatening SVC syndrome and symptomatic bone metastasis before a definitive diagnosis was established. While the thoracic lesion demonstrated sustained radiographic improvement within the radiation field after irradiation, the pelvic bone metastasis progressed shortly after treatment during the same terminal phase. Importantly, serial imaging demonstrated no clear radiographic progression within the thoracic radiation field up to the last available follow‐up, whereas progression occurred outside the irradiated field and at the pelvic metastatic site during the same period (Figure 2).

The thoracic lesion received a substantially higher biologically effective dose than the pelvic lesion, which is the most plausible explanation for the observed difference in radiographic response [2, 3]. Given the marked differences in dose–fractionation and treatment intent between the two sites, no definitive conclusions regarding intrinsic radiosensitivity can be drawn from this comparison.

To date, radiotherapy response patterns in NUT carcinoma have not been systematically evaluated, and available evidence is limited to isolated case reports. Emerging preclinical studies suggest that molecular heterogeneity, including alterations in epigenetic regulators such as *EP300*, may influence treatment sensitivity in NUT carcinoma [4]. Although such molecular factors were not directly assessed in our patient, this observation may be considered hypothesis‐generating; however, it should be interpreted cautiously given the substantial differences in radiotherapy dose and clinical intent between treated sites.

Given the aggressive nature of thoracic NUT carcinoma and its poor prognosis, this case highlights real‐world clinical challenges in managing thoracic NUT carcinoma, including the need for emergent radiotherapy for SVC syndrome before definitive diagnosis and the role of single‐fraction radiotherapy in providing temporary pain palliation for bone metastases. Accumulation of additional cases integrating radiotherapy outcomes with molecular profiling is needed to better understand treatment sensitivity in this rare malignancy.

## Author Contributions

F.K. interpreted the data and drafted the original and revised manuscript. N.T. significantly contributed to data analysis and interpretation. H.K. and K.T. contributed to data analysis and data curation. S.W., H.L., R.O., K.Y., Y.Y., N.K. and H.O. were responsible for data curation. H.K. and T.K. made substantial contributions to revising the manuscript drafts. All authors have reviewed and approved the final version of the manuscript and agree to be accountable for their respective contributions to the work.

## Funding

The authors have nothing to report.

## Consent

The authors declare that written informed consent was obtained for the publication of this manuscript and accompanying images and attest that the form used to obtain consent from the patient complies with the Journal requirements as outlined in the author guidelines.

## Conflicts of Interest

The authors declare no conflicts of interest.

## Data Availability

The data that support the findings of this study are available on request from the corresponding author. The data are not publicly available due to privacy or ethical restrictions.
